# Extra-Legal Contextual Factors, Judge Demographic Characteristics, and the Sentencing of Drug Crimes: Using Pennsylvania Commission of Sentencing Data to Examine Drug Sentencing Severity, 2014–2019

**DOI:** 10.1007/s12103-025-09891-y

**Published:** 2026-01-10

**Authors:** S.H. Davis, M. Kurlychek, A.A. Jones

**Affiliations:** 1 Department Criminology and Sociology, The Pennsylvania State University, University Park, PA 16801 USA; 2 Department of Criminology, Florida State, Tallahassee, FL 32306 USA; 3 Department of Human Development and Family Studies, The Pennsylvania State University, University Park, PA 16801 USA

**Keywords:** Sentencing, Judge, Drug policy, Sex, Race

## Abstract

This study examines how judicial traits (race, sex) and the local court environment influence sentencing decisions for drug offenses, extending beyond the typical focus on individual defendant characteristics. We analyzed 100,652 cases of drug possession and possession with intent to distribute (PWID) from the Pennsylvania Commission on Sentencing (2014–2019). Our dataset comprises 68,268 drug possession cases and 30,116 PWID cases, handled by 519 judges: 76.69% male, 23.31% female, 8.48% minority (with Black judges accounting for 77% of this minority group), and 91.52% White. Using nested OLS and logistic regression, we studied the effect of court setting (rural/urban) and judge demographics on drug sentencing and incarceration, while controlling for the convicted individual’s race, sex, guideline constructs, and prior arrest record. Our findings show that minority male and minority female judges were less likely than White judges to incarcerate individuals convicted with drug possession and PWID. Specifically, a Black judge’s presence lowered the likelihood of incarceration across all racial groups, with a stronger impact on minority defendants. Other factors that influenced sentencing included urban court location, the individual’s race, sex, and past arrest history. These results emphasize the critical role of judge demographics in sentencing decisions and highlight the need for ongoing efforts to ensure fair treatment for all individuals convicted with drug offenses, regardless of the judge’s demographic background.

## Introduction

The proliferation of drug-related arrests and convictions continues to fuel mass incarceration in the United States, raising persistent concerns about racial and social disparities in criminal justice processing. While the enactment of structured sentencing guidelines was intended to reduce unwarranted variation and promote uniformity, outcomes remain influenced by legally irrelevant factors. This is particularly true for drug offenses, where structural policies and charging practices often embed inequality early in the process, creating systemic disparities that persist through sentencing. Critically, much of the existing research focuses narrowly on convicted individual characteristics (e.g., prior record, offense severity) to explain these outcomes, leaving a significant theoretical gap in understanding the role of the primary decision-maker. Building directly upon the focal concerns perspective, which posits that judges’ sentencing decisions are mediated by their subjective perceptions of risk and culpability, this study moves beyond the individual defendant to examine judicial and contextual influences. Utilizing a robust sample of 100,652 cases from the Pennsylvania Commission on Sentencing (2014–2019), we employ hierarchical analysis to examine how judicial demographics (race and sex) and the local court context influence the severity of sentencing for both drug possession and drug dealing offenses. Our findings contribute significantly to the literature by demonstrating that a judge’s demographic background, particularly minority status, is a powerful and often overlooked predictor of sentencing outcomes. We find that minority judges significantly predict lower incarceration rates and, through interaction effects, exhibit distinct sentencing patterns based on the race of the convicted individual, highlighting the critical role of judicial identity in discretionary sentencing.

## Background

### Substance Use

Societal perceptions on the legalization and the criminalization of drugs continue to evolve; however, drug-related offenses still contribute substantially to the nation’s mass incarceration (Lurigio, 2024). Much of the overcriminalization in the U.S. can be attributed to enacted mandatory minimum sentences and, in general, the harsher sentencing of people convicted of drug offenses (Pryor et al., 2017). Pennsylvania, where the present study is based, passed mandatory minimum laws for drug offenses in 1988 that were based on drug amounts in possession; however, in 2015, the Pennsylvania Supreme Court found many aspects of mandatory minimums to be unconstitutional and removed most (Degenhardt et al., 2017).The legislation covered both schedule I (defined “as a high potential for abuse, no currently accepted medical use in the United States, and a lack of accepted safety for use under medical supervision”) and II (defined as “a high potential for abuse, currently accepted medical use in the United States, or currently accepted medical use with severe restrictions, and abuse may lead to severe psychic or physical dependence”) drugs (“The Controlled Substances, Drugs, Device, and Cosmetic Act”, 1972). Moreover, if convicted individuals have a previous drug trafficking offense, the sentencing penalties are raised to 2 years (18 Pa. C.S. 7508). Despite these harsher sentencing laws, drug use and overdose mortality rates in Pennsylvania increased from 8.1 in 1999 to 43.2 in 2021. (CDC, 2022). (Johnson et al., 2021). While there is little to no evidence that mandatory drug sentences have deterred substance use and mortality, evidence suggests that much of the extent of racial disparities in sentencing can be attributed to the enactment of such laws (Alexander, year Painter-Davis & Ulmer, 2020).

While the existing literature addresses racial disparities, a deeper examination reveals that these differences are often structurally embedded through how drug offenses are categorized and prosecuted. Several key studies underscore that the specific substance and the defendant’s role significantly shape sentencing outcomes, independent of individual judicial bias. For instance, Kautt and Spohn (2002) showed that statutory penalties assigned to particular drug types (like the federal crack/powder disparity) were major drivers of overall racial disparity post-guidelines. Similarly, Spohn and Sample (2013) emphasized that these disparities often arise much earlier, at the charging and plea-bargaining stages, when the initial offense severity that dictates the final sentence is determined. Furthermore, the implementation of sentencing reforms, such as mandatory minimums, often fails to eliminate disparity entirely. These findings highlight the necessity of controlling for specific drug types and the severity of the conviction (PWID versus Possession) as central mechanisms through which disparity is created and maintained, a primary aim of the current study.

### Racial Differences in Criminal Justice Outcomes

Much attention to racial disparities in sentencing began during the civil rights movement in the 1960 s and extended into the 1970s. Sentencing guidelines, first introduced in 1982, were mainly developed to reduce disparity in sentencing, and some studies have found slight decreases in racial disparities in sentencing in guideline states (King & Light, 2019; Painter-Davis & Ulmer, 2020). However, many studies still indicate significant racial disparities in arrests, and particularly in drug arrests (Mitchell & Caudy, 2015), convictions (Goldman, 2018) and sentencing decisions (Painter-Davis & Ulmer, 2020) even in states with sentencing guidelines. Overall, racial and ethnic minorities are often given harsher and longer sentences for drug-related crimes (Camplain et al., 2020). Yet, the overrepresentation of racial and ethnic minorities in drug offenses, therefore, cannot be attributed to patterns of use (Camplain et al., 2020).

One reason thought to be the cause of differences in drug offense outcomes is the differing drug types used and offenses convicted between races. One study comparing Black and White people who are convicted of drug offenses found that Black people were more likely to be convicted of possession and sales of simple possession. In contrast, White individuals convicted of drug offenses were more likely to be convicted of illegal activity relating to drugs, even though they reported equal levels of drug sales (Rosenberg et al., 2017). This charge disparity could account for some levels of sentencing disparity. Other studies suggest that the disparity may result from the type of drug used, such as the crack cocaine to powder disparity that was later addressed by the Fair Sentencing Act of 2010 (Tuttle, 2019). However, this study also found that Black persons with a drug use disorder were more likely to prefer marijuana rather than harder drugs and often had less serious drug problems (Rosenberg et al., 2017). While the race of the convicted individual is an imperative aspect in drug sentencing, external factors, such as judge characteristics may influence the sentencing outcomes as well. convicted.

Most studies of sentencing outcomes and particularly disparities in outcomes utilize the focal concerns theoretical perspective. According to this theory, judges balance competing “concerns” at the time of sentencing which include the culpability of the individual convicted, the risk the individual poses to the community, and the practical constraints on sentencing (Galvin & Ulmer, 2021; Steffensmeier et al., 1998). However, the theory outwardly acknowledges that these interpretations may be influenced by judicial stereotypes and biases related to their own race, sex, and court context.

An early study examining Detroit judges showed little predictive power in the judge’s race on sentencing, and that *both* Black and White judges sentence Black individuals with drug-related offenses more harshly than White individuals (Spohn, 1990). Another study examining sentencing decisions in Pennsylvania found that Black and White judges sentenced Black and White defendants similarly (Steffensmeier & Britt, 2001). Similarly, Schanzenbach found that race had no difference among judges’ sentencing decisions (Schanzenbach, 2015). However, contrary to the Detroit study, Black and White judges were likely to send both Black and White convicted individuals to prison rather than offer a community-based sanction (Steffensmeier & Britt, 2001).

Others have argued that conceptual and methodological flaws limit these aforementioned studies (Johnson, 2014). Johnson (2014) addressed this concern by examining two possibilities that potentially explain the minimal influence judge background characteristics have on sentencing. This study found that female and minority judges were less likely to sentence severely (Johnson, 2014). Since this study uses the Pennsylvania Commission on Sentencing data, we use these techniques and results to inform our research questions specifically. Applying similar methodologies, we can examine a larger and more recent data set. Moreover, the previous literature has not specifically examined drug offenses, which have significant implications on how judge characteristics impact sentencing severity.

### Current Study

As such, the present study addresses several pertinent questions. First, it examines whether the race and/or gender of the judge influences the sentencing decision regarding incarceration for individuals convicted of a drug offense. Additionally, it explores whether these judicial characteristics impact the length of sentences given to individuals incarcerated for drug-related offenses. Finally, the study investigates whether the race and/or gender of the judge interacts with the race and/or gender of the convicted individual to influence these sentencing decisions. Ultimately, we hope to answer: Do judges’ race, sex, and court location predict sentencing decisions in drug cases, controlling for legally relevant case characteristics?

## Data & Methods

### Data Source

We use case-specific sentencing data from Common Pleas courts in Pennsylvania to answer our research questions. This data is provided by the Pennsylvania Commission on Sentencing (PCS). Each year, the Commission collects and records all sentencing information within the Commonwealth, with the exception of magistrate courts in Philadelphia. The data is gathered by a web-based reporting system through which counties input online entries. Upon receiving the data, PCS performs data cleaning and validation checks before making the data publicly available.

The unit of analysis for this study is the sentencing event (hereafter referred to as a “case”), as recorded by the Pennsylvania Commission on Sentencing (PCS). The PCS data is structured around the judicial action taken against a convicted individual following a finding of guilt in the Common Pleas Court. Since one individual may be convicted of multiple offenses during a single criminal episode, the analysis focuses exclusively on the Most Serious Offense (MSO) for which the individual was sentenced in that particular judgment. This methodological choice is consistent with the framework of the Pennsylvania Sentencing Guidelines, where the MSO dictates the Offense Gravity Score (OGS) and serves as the primary determinant for the recommended sentencing range. By focusing on the MSO, we isolate the judicial decision relevant to the most severe charge in that criminal episode, thereby ensuring that our analysis of sentencing severity and incarceration likelihood accurately reflects the highest-stakes decision made by the presiding judge in each case.

This data includes felony and misdemeanor offenses committed by those 18 years and older or anyone younger than 18 who were certified to be tried as adults. For these specific analyses, we use data from 2014 to 2019. The data also includes detailed information on the offense type and grade, the individual’s prior record, and demographic information on the individual, including race, gender, age, and county of residence.

Another strength of this data set is that Pennsylvania has used sentencing guidelines since 1982. The purpose of these sentencing guidelines is to reduce discretion and increase sentencing uniformity. According to Ulmer, Bader, and Gault, “sentencing guidelines quantify and standardize sentencing decision criteria (e.g., offense severity and prior record), mandate court consideration of these criteria, and recommend a uniform matrix of sentence ranges” (Ulmer et al., 2008). Pennsylvania is also unique in that it is politically and ethnically diverse, with cities and counties of varying sizes, ranging from rural to urban.

**Fig. 1:**
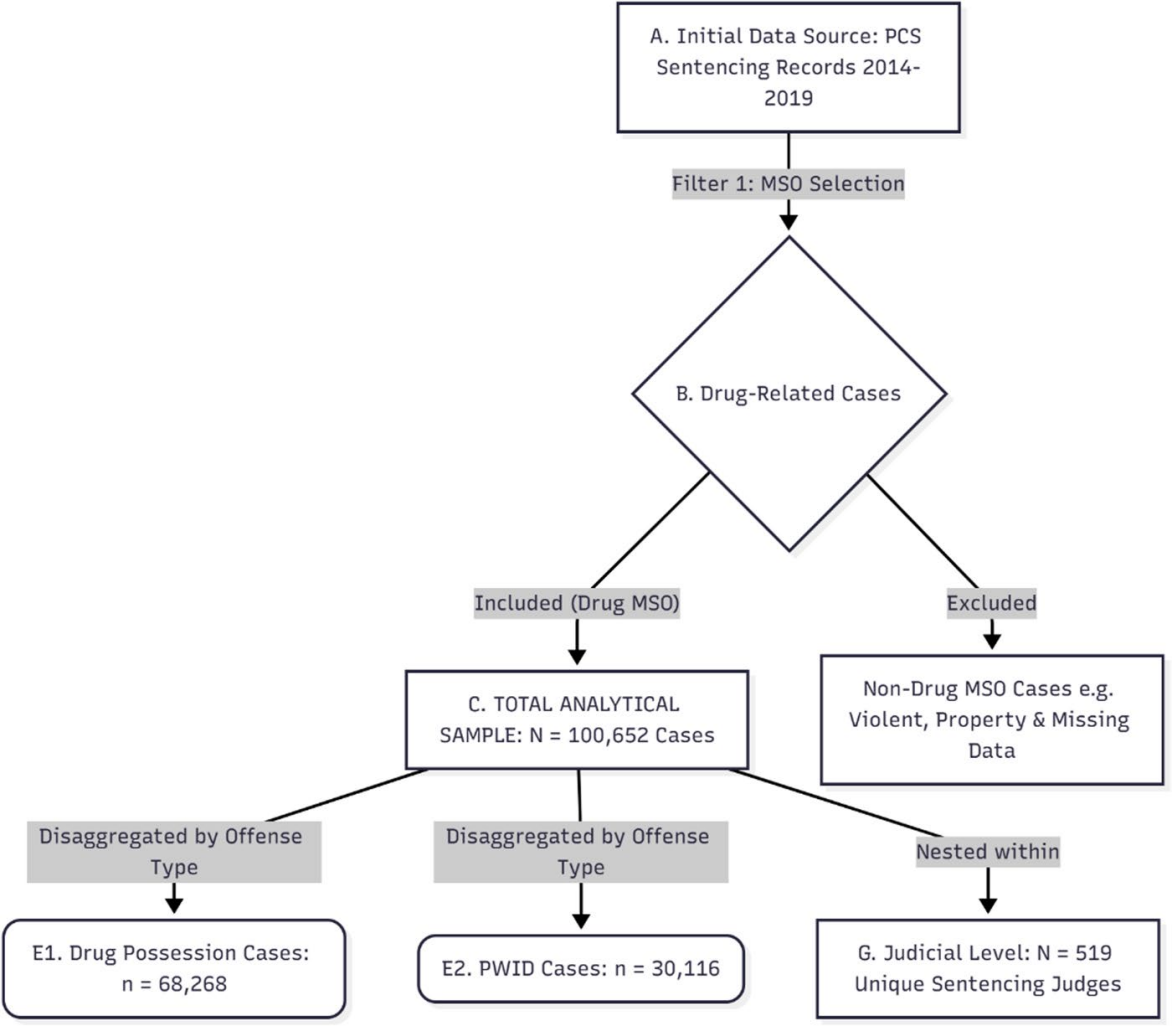
Sampling Flow Diagram

This Fig. 1 illustrates the methodical selection of the analytical sample, beginning with the Pennsylvania Commission on Sentencing (PCS) records from 2014 to 2019. The process applies a critical filter (Filter 1: MSO Selection) to isolate cases where the Most Serious Offense (MSO) was drug-related, excluding non-drug MSOs like violent or property crimes. This filtering yields the Total Analytical Sample of 100,652 cases, which is then disaggregated into two primary subgroups: 68,268 Drug Possession cases and 30,116 Possession With Intent to Distribute (PWID) cases. Finally, the diagram clarifies the hierarchical structure of the analysis, noting that all 100,652 individual cases are nested within 519 unique sentencing judges at the second level of analysis.

### Dependent Variables

The dependent variables relate to sentence punitiveness and type. To understand the impact of judge demographics on sentencing, we examine the decision of whether to incarcerate. The dependent variable of incarceration is a binary variable coded 1 if the convicted individual is sentenced to any length of time in jail or prison and 0 if not. While combining jail and prison is a limitation, we find that most fell into one category (jail). Therefore, the need for separation is less necessary. The second outcome variable of interest is the length of the sentence for those incarcerated. The variable for sentence length is a continuous variable measured by the number of months a convicted individual is sentenced to be incarcerated.

### Independent Variables

The independent variables in this study primarily deal with the characteristics of the judges. Given that the Pennsylvania Commission on Sentencing data lacks judge demographic information, we manually coded the race/ethnicity of the 519 unique judges using verified public sources (judicial directories, campaign records) and employed a double-coding protocol to ensure classification accuracy. We acknowledge that collapsing diverse groups into the “Minority” category (8.48% of judges) risks masking variation; however, due to the small sample size of non-Black minority judges, this aggregation was necessary to produce stable model estimates. A robustness check comparing White, Black, and Other Minority judges confirmed that the significant effect on reduced punitiveness consistently originates from the Black judge subsample. We submit that any residual, non-differential measurement error in the manually coded variable would likely attenuate the statistical effects, suggesting the significant disparities we observe between White and Minority judges are conservative estimates of the true influence of judicial demographics.^1^

The first group of judge characteristics deals with sex and race. Sex is coded as a binary variable with female as ‘0’ and male as ‘1’. The judge’s race is also a binary variable of two separate categories. These categories consist of “White” and “Minority”. The minority category encompasses “Black,” “American Indian,” “Asian/Pacific Island,” “Hispanic,” and “Other.” To understand the intersection of race and gender and their impact on judge decisions, we created a combined categorical variable. The binary variables of race and sex combine to create four categories consisting of “White-male,” “White-female,” “Minority-male,” and “Minority-female.”

While the demographics of the judges may play a role in their decision-making, their location may be just as important. To incorporate locational differences, we include two variables that describe judge location. These variables are visiting judge and county. The visiting judge variable is a binary variable describing whether the judge is a visiting judge in that court for the trial. If the judge is visiting, it is coded as ‘1’ and ‘0’ if they are not visiting. The variable measuring county labeled “urban” is a binary variable that examines whether the judge is practicing in a “Rural” or “Urban” county as defined by the Pennsylvania Commission on Sentencing. If the judge is in an urban county, the data is coded as “1” and “0” if it is a rural county.

### Control Variables

We employ several case-specific factors as controls for precise and accurate results. According to the Pennsylvania sentencing guidelines, the commission data directly measures the convicted individual’s prior criminal record and the current offense’s severity. The Prior Record Score (PRS) quantifies the convicted individual’s previous criminal history. The score consists of eight categories from a PRS of 0 to 5 with two additional categories of repeat violent offender and repeat felony offender. The measure is not a numerical county of prior record but rather considers the severity of offenses. For example, a person could have 1 low level misdemeanor and receive a PRS of 0 but could have only 1 felony offense and receive a PRS of 4. For more information on this scale see appendix.

The Offense Gravity Score (OGS) as coded by PRS ranges from 1 to 14, with 14 being the most serious and 1 being the least serious. By controlling for the OGS and PRS we capture much of the guideline recommendations and ensure that the offense severity nor the individual’s prior record account for judge differentials in sentencing outcomes as these two variables combined determine the recommended sentence as per the guidelines. We also include a categorical variable that identifies if the sentence given was standard, above, or below guideline recommendations, as well as a dichotomous variable on whether the case pleaded out rather than going to trial.

Other control variables we include address the demographics of the convicted individual. These variables include race/ethnicity, gender, and age. Race is coded as 0 for “White” convicted and 1 for “Black” convicted and 2 for all others, including Hispanic, Native American, Asian/pacific islander, and others. The sex of the convicted individual was coded as 0 for female and 1 for male. The last control, the convicted individual’s age, was coded as the age of the convicted individual in years at the time of sentencing.

### Analyses

First, we run descriptive statistics to understand the percentages and averages for each variable. These descriptives are separated for judge, convicted individual, and offense. These statistics help to give a more holistic picture of the frequencies for each typology.

Second, we perform hierarchical multiple regression to further our understanding of the judge characteristic effect on incarceration and length. We estimate the Interclass Correlation Coefficient which indicates that judge random effects compose approximately 25% of the total residual variance. This level of variance at level two (judges) indicates that individual case outcomes are indeed nested within sentencing judges and emphasizes the need for HLM techniques to account for judge variation. For the first dependent variable, the decision to incarcerate or not, we use logistic regression as the outcome is dichotomous. For the second dependent variable, the length of the sentence of incarceration, we use ordinary least squares regression as the dependent variable is continuous. By addressing the in and out verdict and the sentence length within incarceration, we can establish the impact of judge characteristics on the severity of the sentence.

To explore the interaction between judge characteristics and convicted individual characteristics, we create multiplicative variables and present the results using the margins command in Stata to depict the differences between the groups. For the logistic regression models, we use the current best practices prescribed based on our for non-linear link function that involves a second-differencing approach (Long & Mustillo, 2021; Mize, 2019).

## Results

### Descriptives

Among the 128,084 individuals convicted with drug offense, a majority of them are male (77.57%), with an average age of 31.78 years of age. The racial makeup of individuals convicted with drug offenses in Pennsylvania from 2014 to 2019 includes a majority of White (68.8%), with around one-third of Black convicted individuals and all other categories making up around 1%. The mean prior record for those involved in drug crimes is low, around 1.66, and the general gravity score of the offenses is between three and four. Simple possession, along with other drug crimes not specific to a type of drug, make up approximately two-thirds of all drug charges.

Regarding sentencing outcomes, around two-thirds of the individuals convicted were not sentenced to any form of incarceration. However, those sentenced to jail or prison received an average sentence of over 2 years. The median sentence length is 23 months with the lower quartile at 12 months and the upper quartile 24 months.

Given general knowledge of drug crimes and their sentencing outcomes, we must examine the demographics and characteristics of the judges who preside over these cases. The sex distribution for the judges is almost identical to the distribution for the convicted individuals, with over 75% male. While a majority of individuals convicted with drug offense are White, there is a much higher percentage of judges that are White, with non-White judges making up about 6% of the judges. Looking deeper at the location and implications of the culture surrounding these judges is the type of county they work in and represent. The vast majority of judges in these drug cases preside in urban counties. Looking at a more in-depth categorization, slightly over half of all drug cases occurred in the more urban counties (Table 1).

**Table 1:** Descriptive statistics

	*N*	Mean/%	SD	519 judges	*N*	%
*Offender*				Sex		
Sex				Male	398	76.69
female	22,589	22.44		Female	121	23.31
male	78,063	77.56		Race		
Race				White	475	91.52
white	67,823	68.68		Black	34	6.55
black	29,900	30.28		Other	10	1.93
other	1031	1.04		Race & Sex		
Age	100,601	32.99	10.4	white-male	382	73.6
				white-female	93	17.92
*Offense*				black-male	16	3.08
Offense Gravity Score	100,652	3.32	2.36	black-female	18	3.47
Prior Record Score	98,064	1.59	1.87	other-male	3	0.58
Possession	66,859	67.52		other-female	7	1.35
Dealing	32,084	32.48		Visiting judge in the case		
Drug Involved				No	106,095	99.75
Cocaine	7845	7.79		Yes	270	0.25
Designer Drugs	90	0.09		County		
Fentanyl	201	0.2		Urban	93,982	73.85
Heroin	10,856	10.79		Rural	33,275	26.15
MDMA	126	0.13		In-Depth County		
Marijuana	11,555	11.48		More Urban	66,056	52.08
Meth-lab Related	367	0.36		Urban	35,781	28.21
Meth., PCP	1979	1.97		Rural	20,279	15.99
Other Drug Offenses	33,541	33.32		More Rural	4725	3.73
Other Narcotics	2338	2.32		Plea Deal		
Simple Possession	31,745	31.55		Yes	97,285	97.81
Incarcerated				No	2180	2.19
No	68,652	68.21		Guideline Conformity		
Yes	32,000	31.79		Standard	77,070	76.57
				Above	4730	4.7
Length (months)	41,225	27.771	26.736	Below	18,852	18.73

Tables 2 and 3 provide the logistic regression results of the “in and out” decision. This means that if the odds are over one, it is more likely that the convicted individual will be sentenced to incarceration. When looking at the intersection of race and sex of the judges in each case, each group (i.e., White-female, minority-male, etc.) is being compared to a White male judge. With every combination of race and sex, judges are less likely to sentence individuals convicted with drug offenses to incarceration when compared to White male judges.

**Table 2:** Logistic regression incarceration for possession

*N* = 68,268	Odds Ratio	SE	95% CI		*P*
ICC = 0.265379			LL	UL	
Level 1: Judge					
Judge Race & Sex					
white-female	1.023	0.166	0.739	1.414	0.893
minority-male	0.413	0.378	0.197	0.865	0.019
minority-female	0.364	0.332	0.190	0.696	0.002
County					
Urban	0.641	0.129	0.497	0.826	0.001
Visiting judge	0.984	0.325	0.521	1.860	0.961
Level 2: Individual					
Offender Race					
black	1.111	0.033	1.042	1.185	0.001
other	1.350	0.170	0.967	1.886	0.078
Offender Male	1.014	0.034	0.949	1.084	0.674
Prior Record Score (reference category 0)					
1	2.713	0.051	2.453	3.001	0.000
2	2.693	0.065	2.371	3.057	0.000
3	2.410	0.086	2.037	2.850	0.000
4	8.193	0.090	6.869	9.772	0.000
5	10.358	0.102	8.485	12.644	0.000
Offense Gravity Score (reference category 1)					
3	3.963	0.050	3.590	4.376	0.000
Plea Deal	0.569	0.128	0.442	0.732	0.000
Conform to Sentencing Guide (reference category conform)					
Below	0.123	0.066	0.108	0.140	0.000
Above	217.175	0.072	188.676	249.979	0.000
Year	0.972	0.010	0.953	0.993	0.007
Age	1.029	0.009	1.010	1.047	0.002
Prior Record * Offense Gravity Score	1.162	0.009	1.141	1.184	0.000
Age Squared	0.999	0.000	0.999	1.000	0.000
Constant	7.921E + 22	21.106	85738.736	7.317E + 40	0.012

**Table 3:** Logistic regression incarceration for drug dealing

	Odds Ratio	SE	95% CI		*P*
*N* = 30,116			LL	UL	
ICC = 0.2543335					
Level 1: Judge					
Judge Race & Sex					
white-female	1.048	0.165	0.758	1.450	0.776
minority-male	0.499	0.331	0.260	0.955	0.036
minority-female	0.718	0.284	0.411	1.253	0.243
County					
Urban	0.666	0.132	0.514	0.864	0.002
Visiting judge	1.168	0.408	0.526	2.598	0.703
Level 2: Individual					
Offender Race					
black	1.331	0.036	1.240	1.428	0.000
other	1.331	0.131	1.029	1.720	0.029
Offender Male	1.525	0.041	1.407	1.653	0.000
Prior Record Score (reference category 0)					
1	3.269	0.060	2.906	3.676	0.000
2	6.027	0.076	5.192	6.997	0.000
3	11.018	0.105	8.974	13.528	0.000
4	21.137	0.130	16.379	27.277	0.000
5	52.702	0.152	39.095	71.045	0.000
6	160.044	1.173	16.053	1595.560	0.000
Offense Gravity Score (reference category 3)					
5	3.014	0.082	2.565	3.540	0.000
6	7.732	0.102	6.332	9.441	0.000
7	11.762	0.106	9.563	14.468	0.000
8	15.894	0.112	12.751	19.812	0.000
9	16.226	0.327	8.551	30.788	0.000
10	32.393	0.151	24.112	43.518	0.000
11	38.830	0.155	28.684	52.564	0.000
13	61.617	0.235	38.865	97.688	0.000
14		(empty)			
Drug Type (Reference Category Cocaine)					
Fentanyl	1.356	0.286	0.775	2.373	0.286
Heroin	1.055	0.055	0.948	1.175	0.325
MDMA	0.548	0.267	0.325	0.923	0.024
Marijuana	0.577	0.077	0.497	0.671	0.000
Meth or PCP	1.111	0.082	0.946	1.304	0.198
Other	0.780	0.063	0.689	0.884	0.000
Other Narcotics	0.561	0.077	0.482	0.653	0.000
Plea Deal	0.886	0.090	0.743	1.058	0.181
Conform to Sentencing Guide (reference category conform)					
Below	0.181	0.041	0.167	0.196	0.000
Above	1.949	0.080	1.665	2.281	0.000
Year	0.974	0.012	0.951	0.998	0.035
Age	1.026	0.009	1.008	1.045	0.005
Prior Record * Offense Gravity Score	0.955	0.005	0.947	0.964	0.000
Age Squared	0.999	0.000	0.999	1.000	0.000
Constant	1.65032E + 22	24.893	10.683	2.54946E + 43	0.040

Looking at drug possession offenses (Table 2), minority judges are significantly less likely to sentence convicted individuals to incarceration. Minority male (OR 0.41, 95% CI: 0.19–0.87) and female (OR 0.36, 95% CI: 0.19–0.69) judges are over 50% less likely to sentence a defendant to jail or prison time compared to White male judges. Judges in an urban county are 35.9% less likely (OR 0.641, 95% CI: 0.49–0.83) to incarcerate possession convicted individuals. There is no significant difference in the impact of the visiting judge’s status on the in-and-out decision regarding possession.

Expanding into the level two variables, we see that race has a significant relationship with the in-and-out decision for possession of drugs. First, examining the convicted individual’s characteristics reveals that if they are non-White, they are more likely to be sentenced to jail or prison. Black convicted individuals’ odds of incarceration are 11% more likely than White convicted individuals (OR 1.11, 95% CI: 1.04–1.19). Age of the convicted individual is positively correlated, meaning the older the individual, the more likely they are to be sentenced to incarceration. However, with years of sentence, we find that the later the individual was sentenced, the less likely they were to be incarcerated.

Examining the characteristics of current and prior offenses reveals that a prior record score is positively correlated with the likelihood of incarceration. The higher the prior record score, the higher the odds of incarceration. For possession, each categorical increase in prior record score increases the odds of being incarcerated for possession. This also holds true for offense gravity score. Cases that ended in a plea deal were 43% less likely to end in incarceration.

Table 3 presents the logistic regression results for PWID (possession with intent to distribute) offenses. Like Table 2, this refers to the “in and out” decision of whether or not to incarcerate. Judge race and sex outcomes are similar for PWID as they were for possession. Again, minority judges are significantly less likely to sentence convicted individuals to incarceration. Minority male judges are 50.1% less likely (OR 0.499, 95% CI:0.26–0.96), while minority female judges are not significantly less likely to sentence PWID to incarceration. Similar to possession crimes, there is a significant effect on whether the judge and case are in an urban versus rural county. Judges in an urban county are 33.4% less likely (OR 0.666, 95% CI: 0.51–0.86) to incarcerate PWID convicted individuals.

Convicted individual characteristics for PWID charges significantly impact the likelihood of their incarceration. Black and minority convicted individuals are around 33.1% more likely (OR 1.331, 95% CI:1.24–1.43; OR 1.331, 95% CI:1.029–1.72) to be incarcerated. Being male, a convicted individual, increases their likelihood of incarceration by 52.5% (OR 1.525, 95% CI: 1.407–1.653). Higher prior record and offense gravity scores are associated with a greater likelihood of imprisonment for PWID.

The impact of drug type on incarceration was only significant when decreasing the drug type reduced the likelihood of incarceration compared to PWID cocaine. MDMA, Marijuana, and other drugs or narcotics are 22–55% less likely to lead to incarceration when compared to cocaine. Unlike possession, plea deals do not have a significant impact on incarceration likelihood.

While looking at the initial in-and-out decision in sentencing is crucial, it is also essential to address the length of time applied to those sentenced to incarceration. We use hierarchical linear OLS regression to understand the correlation between judge characteristics and incarceration length. The results of this regression can be found in Tables 4 and 5. The results for incarceration length are less consistent when looking at judge characteristics.

Examining drug possession crimes, the length of incarceration for possession is not significant for judges. Minority female judges are the closest in significance and are more likely to give longer sentences for drug offenses than White male judges. Urban judges were also more likely to sentence drug possession offenders to more extended incarceration periods than rural judges.

Looking at the controls, the significance of the convicted individuals’ race is concentrated on Black convicted individuals. If the convicted individual is Black, they are more likely to get a shorter sentence than White convicted individuals for possession. This could be that White defendants are less likely to get sentenced compared to Black defendants, so when they are sentenced to jail, it’s for more serious offenses. Male convicted individuals were less likely to receive a longer sentence compared to female drug possession convicted individuals. Prior record and offense gravity scores are positively correlated with incarceration length. Plea deals are negatively correlated with the length of incarceration. As with the in-and-out decision, the later in the year the individual was sentenced, the shorter the sentence (Table 4).

**Table 4:** OLS regression incarceration length for possession

*N* = 11,358	Coefficient	SE	95% CI		*P*
ICC = 0.2005315			LL	UL	
Level 1: Judge					
Judge Race & Sex					
white-female	0.197	0.442	−0.669	1.063	0.656
minority-male	0.929	1.120	−1.266	3.123	0.407
minority-female	1.224	0.976	−0.690	3.138	0.210
County					
Urban	0.828	0.346	0.149	1.507	0.017
Visiting judge	−0.012	1.212	−2.388	2.364	0.992
Level 2: Individual					
Offender Race					
black	−1.249	0.121	−1.485	−1.012	0.000
other	−0.946	0.592	−2.106	0.214	0.110
Offender Male	−0.486	0.128	−0.737	−0.236	0.000
Prior Record Score (reference category 0)					
1	1.602	0.224	1.163	2.041	0.000
2	1.548	0.270	1.019	2.077	0.000
3	0.901	0.336	0.242	1.561	0.007
4	1.317	0.388	0.558	2.077	0.001
5	1.675	0.443	0.806	2.544	0.000
Offense Gravity Score (reference category 1)					
3	4.818	0.199	4.427	5.209	0.000
Plea Deal	−1.047	0.421	−1.873	−0.221	0.013
Conform to Sentencing Guide (reference category conform)					
Below	−3.410	0.194	−3.791	−3.029	0.000
Above	2.482	0.201	2.088	2.875	0.000
Year	−0.101	0.039	−0.177	−0.025	0.009
Age	0.143	0.035	0.075	0.211	0.000
Prior Record * Offense Gravity Score	0.369	0.031	0.308	0.430	0.000
Age Squared	−0.002	0.000	−0.003	−0.001	0.000
Constant	206.533	77.864	53.922	359.143	0.008

Table 5 presents the results of an ordinary least squares regression of incarceration length for PWID charges. When we examine incarceration length for individuals convicted of possession with intent to deliver, judge race and sex are significant across all minority categories. Both minority male and female judges sentence PWID offenders to shorter sentences than white male judges. Similar to possession, judges in urban counties are significantly more likely to sentence individuals facing these charges to shorter incarceration lengths than their rural counterparts.

**Table 5:** OLS regression incarceration length for drug dealing

*N* = 19,042	Coefficient	SE	95% CI		*P*
ICC = 0.1110029			LL	UL	
Level 1: Judge					
Judge Race & Sex					
white-female	−0.427	1.060	−2.504	1.650	0.687
minority-male	−4.715	2.283	−9.190	−0.240	0.039
minority-female	−5.053	1.882	−8.742	−1.365	0.007
County					
Urban	−4.014	0.853	−5.687	−2.341	0.000
Visiting judge	−0.775	3.119	−6.888	5.337	0.804
Level 2: Individual					
Offender Race					
black	0.282	0.316	−0.337	0.901	0.372
other	0.935	1.051	−1.125	2.996	0.374
Offender Male	1.150	0.414	0.340	1.961	0.005
Prior Record Score (reference category 0)					
1	0.436	0.558	−0.658	1.530	0.434
2	1.181	0.674	−0.139	2.502	0.080
3	3.019	0.905	1.246	4.792	0.001
4	3.089	1.102	0.930	5.249	0.005
5	7.884	1.292	5.352	10.416	0.000
6	18.177	6.573	5.295	31.060	0.006
Offense Gravity Score (reference category 3)					
5	5.971	0.948	4.113	7.828	0.000
6	13.762	1.064	11.676	15.849	0.000
7	17.008	1.095	14.862	19.155	0.000
8	20.420	1.148	18.171	22.670	0.000
9	27.794	2.800	22.306	33.283	0.000
10	50.381	1.393	47.650	53.111	0.000
11	58.021	1.447	55.185	60.858	0.000
13	86.231	1.952	82.404	90.057	0.000
14	111.035	6.650	98.001	124.069	0.000
Drug Type (Reference Category Cocaine)					
Fentanyl	3.401	2.089	−0.693	7.494	0.103
Heroin	0.635	0.433	−0.213	1.484	0.142
MDMA	−3.971	2.371	−8.618	0.675	0.094
Marijuana	0.271	0.824	−1.344	1.887	0.742
Meth or PCP	0.296	0.611	−0.901	1.494	0.628
Other	−1.422	0.630	−2.657	−0.187	0.024
Other Narcotics	−3.865	0.675	−5.188	−2.542	0.000
Plea Deal	−8.460	0.649	−9.733	−7.188	0.000
Conform to Sentencing Guide (reference category conform)					
Below	−20.123	0.346	−20.800	−19.446	0.000
Above	28.980	0.552	27.898	30.063	0.000
Year	−0.285	0.107	−0.495	−0.075	0.008
Age	0.117	0.091	−0.061	0.295	0.199
Prior Record * Offense Gravity Score	0.680	0.038	0.606	0.754	0.000
Age Squared	−0.002	0.001	−0.004	0.001	0.188
Constant	593.366	216.162	169.696	1017.035	0.006

The level two variables of convicted individual sex, offense gravity, and prior record are all significant, while the offender’s race is not significant. However, males and individuals with a higher prior record or offense gravity scores were more likely to be sentenced to a longer period in jail or prison. Consistent with all other tables, year is negatively correlated while age is positively correlated with sentence length. Plea deals result in significantly shorter incarceration stays.

Finally, we explore the possibility that judge characteristics may interact with convicted individual characteristics in determining sentencing outcomes. Our results reveal significant variances in the likelihood of incarceration based on the interaction of judge and convicted individual race and sex for both possession and possession with intent to deliver charges. For possession, results presented in Appendix A, having a Black judge reduces the likelihood of incarceration by 11% for White convicted individuals (*p* =.000), by 15.8% for Black convicted individuals (*p* =.000), and 19.8% for other convicted individuals (*p* =.056, marginally significant). This same pattern holds for possession with intent to deliver with the likelihood of incarceration reduced for all convicted individuals but only achieving significance for Black (13.1%, *p* =.006) and other convicted individuals (16.6%, *p* =.047). While judges of other races are also less likely to sentence convicted individuals to incarceration than White judges are, these findings do not reach statistical significance. Regarding gender, results presented in Appendix B, having a male judge reduces the likelihood of incarceration for possession by 4% when the convicted individual is female (*p* =.08). This is marginally significant and there are no other significant interactions for sex.

## Discussion

The findings from this study highlight the critical role that judge characteristics can play in sentencing outcomes for drug offenses. In particular, the judge’s race and sex seem to be significant factors. White male judges were consistently found to be more severe in their sentencing of individuals convicted of drug offenses compared to judges of other race/sex combinations. Minority male and female judges were the least likely to sentence individuals convicted with drug offense to incarceration compared to their White male counterparts. Recalling the focal concerns theory, a judge’s decision-making is often influenced by the convicted individual’s risk assessment and culpability. Thus, this could signify that different races and genders perhaps perceive these factors differently. It could also be that White males are a majority and ruling class in society, see those of other races as more of a threat than those judges that themselves fall into some sort of minority or oppressed status (Shelton et al., 2006). Recalling the focal concerns theory, a judge’s decision-making is often influenced by the convicted individual’s risk assessment and culpability (Galvin & Ulmer, 2021). Since the judges decide how these two aspects impact sentencing, their own views and biases, specifically against race and gender, can influence their discretion. Thus, the consistent finding that Minority judges (predominantly Black) are significantly less punitive signifies that they interpret these core concerns through a different lens.

These disparities raise concerns about potential biases and unwarranted sources of variation in how the law is applied. While sentencing guidelines promote uniformity, the results suggest that extralegal factors related to the judge may still contribute to the disparate treatment of convicted individuals. This underscores the difficulty of removing human discretion entirely from the judicial process.

The context of the local community also appeared to matter, with judges in rural areas sentencing individuals convicted of a drug offense, particularly PWID convicted individuals, more harshly in terms of incarceration likelihood and sentence length compared to urban judges. This could reflect differences in social and political environments across communities that shape judicial philosophies and attitudes toward drug crimes. It also could reflect differences in caseload noted previously. With urban areas subject to larger caseloads, it could be that the practical constraints (a.k.a. focal concerns theory) overwhelm the court, and the jail/prison capacity becomes a factor. Also, we know that in Pennsylvania, urban areas tend to lean more liberally and rural areas more conservatively, which may also influence the sentencing severity of judges in these areas.

It is concerning that the convicted individual’s race remains a significant predictor of sentencing outcomes even after controlling for offense severity and prior records. Racial minorities, especially Black convicted individuals, faced higher odds of incarceration for drug possession and possession with intent to deliver compared to their White counterparts. For possession offenses, Black convicted individuals received shorter sentence lengths, perhaps due to the overuse of imprisonment in general for these crimes among this group. Nevertheless, the persistent racial disparities call into question the equitable application of justice.

Regarding the interactions between convicted individual race and judge race, the most consistent pattern found was that the mitigating effect of a Black judge on the likelihood of incarceration was substantially stronger for Black and other minority convicted individuals compared to White convicted individuals (See Appendix A). This crucial finding supports the existence of complex in-group/out-group dynamics in sentencing literature (Johnson, 2006). This could signify that, overall, Black judges are more cognizant of individuals convicted with drug offense requiring treatment rather than punishment, as this is the population that has been most impacted by the “war on drugs.” Furthermore, it signals that Black judges may not attribute as much riskiness and/or culpability to other Black and minority convicted individuals and may be more sympathetic to their plight (Shelton et al., 2006), ultimately leading to a narrower racial disparity gap in sentencing outcomes compared to courts presided over by White judges.

The context of the local community also appeared to matter, with judges in rural areas sentencing individuals convicted of a drug offense, particularly PWID convicted individuals, more harshly in terms of incarceration likelihood and sentence length compared to urban judges. This could reflect differences in social and political environments across communities that shape judicial philosophies and attitudes toward drug crimes. It also could reflect differences in caseload noted previously. With urban areas subject to larger caseloads, it could be that the practical constraints (a.k.a. focal concerns theory) overwhelm the court, and the jail/prison capacity becomes a factor. Also, we know that in Pennsylvania, urban areas tend to lean more liberally and rural areas more conservatively, which may also influence the sentencing severity of judges in these areas.

It is concerning that the convicted individual’s race remains a significant predictor of sentencing outcomes even after controlling for offense severity and prior records. Racial minorities, especially Black convicted individuals, faced higher odds of incarceration for drug possession and possession with intent to deliver compared to their White counterparts. For possession offenses, Black convicted individuals received shorter sentence lengths, perhaps due to the overuse of imprisonment in general for these crimes among this group. Nevertheless, the persistent racial disparities call into question the equitable application of justice.

### Limitations

Despite the robust sample size and the use of Hierarchical Linear Modeling (HLM), this study is subject to several limitations inherent in the analysis of administrative sentencing data. First, we combined all forms of incarceration (jail and prison) into a single outcome measure. Although the majority of individuals sentenced for these offenses in Pennsylvania are sentenced to county jail, combining these categories masks potential differences in judicial decision-making regarding the type of incarceration and its implied severity. Future research should prioritize separating these outcomes to refine the findings related to sentence length.

Second, the administrative nature of the Pennsylvania Commission on Sentencing (PCS) data limits our ability to control for critical case-level factors that occur before the sentencing hearing. Specifically, the data lacks information on prosecutor recommendations, which are a powerful predictor of final sentence outcomes, and does not capture the details of the plea bargaining process that precedes conviction. The inability to fully account for these pre-sentencing influences means that some variation attributed to the judge may, in fact, be influenced by prosecutorial charging and negotiation strategies.

## Conclusion

In summary, this study reveals essential judge-based and contextual sources of disparity in drug sentencing within a structured guideline state. The persistent disparities, particularly the significant protective effect of minority judges, demonstrate that the implementation of guidelines alone is insufficient to eliminate extralegal influences. These results argue strongly for policies that prioritize judicial workforce diversity as a necessary component of sentencing reform efforts aimed at achieving fair treatment and equity. Given the long history of racial discrimination surrounding drug policy enforcement in America, these findings are highly problematic. They demonstrate that work remains to eliminate extralegal influences and ensure true fairness and consistency across the criminal justice system. Furthermore, continuing research must move beyond simple comparisons and utilize hierarchical models to disentangle these complex interaction effects between the identity of the judge and the convicted individual to fully understand discretionary decision-making.

Addressing these issues requires a combination of efforts, including enhanced judicial training, accountability measures, representative workforce diversity, and re-examining the appropriate roles of judicial discretion versus rigid guidelines. Notably, the results remind us that discretionary decision-makers do not operate in a vacuum - their personal perspectives and environmental contexts can shape consequential outcomes. Continuing research in this area remains critical for identifying sources of disparity and potential solutions. 

## Appendix A: Interaction Tables for Race of Judge and Race of Convicted Individual

**Table 6:** Incarceration decision for possession

Possession					
2nd difference for In and out decision					
	Delta-method				
	dy/dx	SE	95% CI		P
			[LL	UP]	
White Judge	(base outcome = white judge)				
Black Judge					
Convicted Individual race					
White	−0.113	0.032	−0.176	−0.050	0.000
Black	−0.158	0.034	−0.224	−0.092	0.000
Other	−0.198	0.104	−0.401	0.005	0.056
Other Judge					
Convicted Individual race					
White	−0.067	0.050	−0.164	0.031	0.181
Black	−0.061	0.060	−0.178	0.056	0.308
Other	−0.057	0.108	−0.268	0.155	0.599

**Table 7:** Incarceration decision, PWID

Dealing					
2nd difference for in and out decision					
	Delta-method				
	dy/dx	SE	95% CI		P
			[LL	UL]	
White Judge	(base outcome = white judge)				
Black Judge					
Offrace					
White	−0.073	0.053	−0.176	0.031	0.170
Black	−0.131	0.048	−0.224	−0.037	0.006
Other	−0.166	0.083	−0.329	−0.002	0.047
Other Judge					
Convicted Individual race					
white	−0.082	0.077	−0.232	0.069	0.287
black	−0.015	0.060	−0.132	0.103	0.807
other	−0.204	0.130	−0.460	0.051	0.117

**Table 8:** Length decision, possession

Incarceration Length					
	Delta-method				
	dy/dx	SE	95% CI		P
			[LL	UP]	
White Judge	(base outcome)				
Black Judge					
Convicted Individual race					
white	2.429	1.301	−0.121	4.979	0.062
black	2.294	1.346	−0.344	4.933	0.088
other	−4.090	6.383	−16.601	8.421	0.522
Other Judge					
Convicted Individual race					
white	−0.818	1.431	−3.623	1.987	0.568
black	1.627	1.555	−1.420	4.674	0.295
other	1.175	2.988	−4.681	7.031	0.694

**Table 9:** Length Decision, PWID

Incarceration Length					
	Delta-method				
	dy/dx	SE	95% CI		P
			[LL	UL]	
white judge	(base outcome)				
black judge					
Convicted Individual race					
white	−9.846	3.121	−15.964	−3.729	0.002
black	−11.988	2.858	−17.591	−6.385	0.000
other	−11.952	5.449	−22.631	−1.272	0.028
Other judge					
Convicted Individual race					
white	−2.185	4.287	−10.587	6.217	0.610
black	−1.666	4.180	−9.858	6.527	0.690
other	−3.401	7.293	−17.696	10.894	0.641

**Table 10:** Crosstabulations

Judge-Offender Race Crosstabulation				
	Offender Race			
Judge Race	white	black	other	Total
black	1,603	1,395	78	3,076
other	2,042	838	72	2,952
white	64,178	27,667	881	92,726
Total	67,823	29,900	1,031	98,754

## Appendix B: Interaction Tables for Sex of Judge and Sex of Convicted Individual

**Table 11:** Incarceration decision for possession

Possession					
In and out					
	Delta-method				
	dy/dx	SE	95% CI		P
			[LL	UL]	
Female Judge	(base outcome)				
Male Judge					
Convicted Individual sex					
female	−0.042	0.024	−0.089	0.005	0.080
male	0.006	0.022	−0.036	0.049	0.768

**Table 12:** Incarceration decision for PWID

PWID					
In and Out					
	Delta-method				
	dy/dx	SE	95% CI		P
			[LL	UL]	
Female Judge	(base outcome)				
Male Judge					
Convicted Individual sex					
female	0.023	0.035	−0.046	0.092	0.509
male	0.010	0.023	−0.035	0.056	0.655

**Table 13:** Length Decision, possession

Incarceration length					
	Delta-method				
	dy/dx	SE	95% CI		P
			[LL	UL]	
Female Judge	(base outcome)				
Male Judge					
Convicted Individual sex					
female	−0.348	0.603	−1.530	0.835	0.564
male	−0.177	0.502	−1.161	0.807	0.724

**Table 14:** Length Decision, PWID

Incarceration Length					
	Delta-method				
	dy/dx	SE	95% CI		P
			[LL	UL]	
Female Judge	(base outcome)				
Male Judge					
Convicted Individual sex					
female	3.503819	2.207	−0.823	7.830	0.112
male	1.094039	1.539	−1.922	4.110	0.477

**Table 15:** Crosstabulation

Judge-Offender Sex Crosstabulation			
	Offender Sex		
Judge Sex	Female	Male	Total
Female	4,756	17,093	21,849
Male	17,833	60,970	78,803
Total	22,589	78,063	100,652

## Footnotes

1 Judicial race/ethnicity was determined by reviewing publicly available information, including census data, professional biographies, awards, and other relevant sources. Judges were categorized as White, Black, or Other based on this collected data.
